# Robust and simplified machine learning identification of pitfall trap‐collected ground beetles at the continental scale

**DOI:** 10.1002/ece3.6905

**Published:** 2020-11-11

**Authors:** Jarrett Blair, Michael D. Weiser, Michael Kaspari, Matthew Miller, Cameron Siler, Katie E. Marshall

**Affiliations:** ^1^ Department of Zoology University of British Columbia Vancouver BC Canada; ^2^ Department of Biology University of Oklahoma Norman OK USA; ^3^ Sam Noble Oklahoma Museum of Natural History University of Oklahoma Norman OK USA

**Keywords:** Carabidae, computer vision, insect sampling, machine learning, macroecology

## Abstract

1. Insect populations are changing rapidly, and monitoring these changes is essential for understanding the causes and consequences of such shifts. However, large‐scale insect identification projects are time‐consuming and expensive when done solely by human identifiers. Machine learning offers a possible solution to help collect insect data quickly and efficiently.
2. Here, we outline a methodology for training classification models to identify pitfall trap‐collected insects from image data and then apply the method to identify ground beetles (Carabidae). All beetles were collected by the National Ecological Observatory Network (NEON), a continental scale ecological monitoring project with sites across the United States. We describe the procedures for image collection, image data extraction, data preparation, and model training, and compare the performance of five machine learning algorithms and two classification methods (hierarchical vs. single‐level) identifying ground beetles from the species to subfamily level. All models were trained using pre‐extracted feature vectors, not raw image data. Our methodology allows for data to be extracted from multiple individuals within the same image thus enhancing time efficiency, utilizes relatively simple models that allow for direct assessment of model performance, and can be performed on relatively small datasets.
3. The best performing algorithm, linear discriminant analysis (LDA), reached an accuracy of 84.6% at the species level when naively identifying species, which was further increased to >95% when classifications were limited by known local species pools. Model performance was negatively correlated with taxonomic specificity, with the LDA model reaching an accuracy of ~99% at the subfamily level. When classifying carabid species not included in the training dataset at higher taxonomic levels species, the models performed significantly better than if classifications were made randomly. We also observed greater performance when classifications were made using the hierarchical classification method compared to the single‐level classification method at higher taxonomic levels.
4. The general methodology outlined here serves as a proof‐of‐concept for classifying pitfall trap‐collected organisms using machine learning algorithms, and the image data extraction methodology may be used for nonmachine learning uses. We propose that integration of machine learning in large‐scale identification pipelines will increase efficiency and lead to a greater flow of insect macroecological data, with the potential to be expanded for use with other noninsect taxa.

## Graphical abstract

We outline a methodology for training classification models to identify pitfall trap‐collected insects from image data and then apply the method to identify ground beetles (Carabidae). The best performing model reached an accuracy of 84.6% at the species level, which was further increased to >95% when classifications were limited by known local species pools.

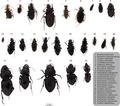

## 1 INTRODUCTION

Insects have an irreplaceable role in ecosystem functioning as they are key contributors to processes such as nutrient cycling and pollination (Gullan & Cranston, 2014). However, some studies have shown recent declines in insect abundance and biomass (Hallmann et al., 2017; Tseng et al., 2018; Welti et al., 2020; Wepprich et al., 2019). For example, recent estimates suggest global terrestrial insect populations alone are decreasing by ~9% per decade (van Klink et al., 2020). Currently, it remains unclear whether these declines reflect: (a) decreasing insect population sizes, (b) decreasing insect diversity, and/or (c) reductions in insect body sizes in response to climate change (Angilletta & Dunham, 2003; Tseng et al., 2018). However, despite these alarming observations, we continue to lack efficient means for surveying, identifying, and monitoring global insect populations. Therefore, it is imperative that we invest in improving insect population monitoring techniques so we can better understand the causes, potential outcomes, and solutions for insect population declines (Thomas et al., 2019).

### 1.1 The NEON monitoring network

One project looking to develop long‐term monitoring capabilities is the National Ecological Observatory Network (NEON), which gathers and provides open access ecological data at a continental scale in the United States (Keller et al., 2008). NEON researchers collect data on weather, land use, biogeochemistry, ecohydrology, and community composition at research stations throughout the entire United States including Puerto Rico (Thorpe et al., 2016). NEON’s long‐term ecological monitoring goals are to understand and forecast the effects of climate change, land‐use change, and invasive species on ecosystems (Thorpe et al., 2016). As part of their ecological monitoring, NEON collects and processes thousands of invertebrate specimens every year from an array of pitfall traps that are run for 14 days to estimate local abundance and diversity of ground beetles (Coleoptera: Carabidae; see Hoekman et al., 2017; Thorpe et al., 2016). The current workflow for NEON carabid sampling is for a local technician/parataxonomist to collect the samples and then separate and identify all adult carabid beetles. Some samples are then sent on to expert taxonomists to confirm identifications. This data are then uploaded to NEON’s open access data portal for use in scientific research (NEON, 2020).

Although NEON has made this standardized carabid beetle data accessible to ecologists, manually sorting, identifying, and counting these specimens is time‐consuming, labor‐intensive, and costly (Karlsson et al., 2020). This can create backlogs of specimens spanning years or decades and limit large‐scale projects to nations with access to the necessary funding (Karlsson et al., 2020). In order for insect monitoring data to be useful for making conservation management decisions, the rate at which we process and analyze samples must keep up with the rate at which insect populations are changing (Lindenmayer et al., 2012). Expanding access of large‐scale monitoring projects to developing nations will also be required to properly make generalizations of global insect population changes (van Klink et al., 2020).

### 1.2 Developing machine learning models to expand monitoring

Machine learning is a tool potentially useful for insect monitoring (Thessen, 2016). One of the most common uses of machine learning in ecology is the identification of organisms (including insects) from images (MacLeod et al., 2010), where it can quickly and accurately identify invertebrates to species (Ärje et al., 2020; Ding & Taylor, 2016; Marques et al., 2018; Mayo & Watson, 2007). In this study, we use NEON’s vouchered carabid collection as a proof‐of‐concept to ask whether machine learning can be employed to better automate and expedite the identification of invertebrates from vouchered community samples while also collecting relevant morphometric data.

Machine learning is not without its own challenges. To be effective, many algorithms, like those from mobile applications like iNaturalist and Plantix, require thousands (if not millions) of images (Van Horn et al., 2018). The way these projects currently amass such datasets is by utilizing large‐scale citizen science efforts, which are typically biased toward charismatic species (Chase & Levine, 2016). Moreover, machine learning models trained primarily using living specimens may not be suitable for identifying dead specimens that have deteriorated over time. For many researchers who wish to use machine learning, these barriers are not possible to overcome.

One solution is to train machine learning classification models from image feature vectors, not from raw pixel data (Joutsijoki et al., 2014; Kho et al., 2017; Larios et al., 2008; Mayo & Watson, 2007). Feature vectors are comprised of descriptive variables such as specimen size, shape, and color that can be extracted from open access programs like ImageJ (Schneider et al., 2012). Models using pre‐extracted feature vectors are a potential improvement over using raw image data, as their mechanisms (e.g., variable importance) are more transparent compared to more algorithms like convolutional neural networks (Archer & Kimes, 2008). Here, we expand on previous studies—all of which use images containing a single individual only—and increase their utility to monitoring studies by generating feature vectors from images of multiple individuals that might be caught in a single trapping event. Likewise, rather than focus solely on species‐level identifications (IDs), which are not required nor realistic for many entomological monitoring programs (Lenat & Resh, 2001), we use an hierarchical classifier to build vector‐trained models that incorporate taxonomic resolution (Gordon, 1987). An hierarchical classifier exploits the nested nature of evolution to identify a specimen at multiple taxonomic levels without the need to train additional models. For example, if the base classifier can make predictions at the species level, an hierarchical classifier would be able to use those predictions to make classifiers at all higher taxonomic levels (i.e., genus, tribe, family, etc.).

**FIGURE 1:**
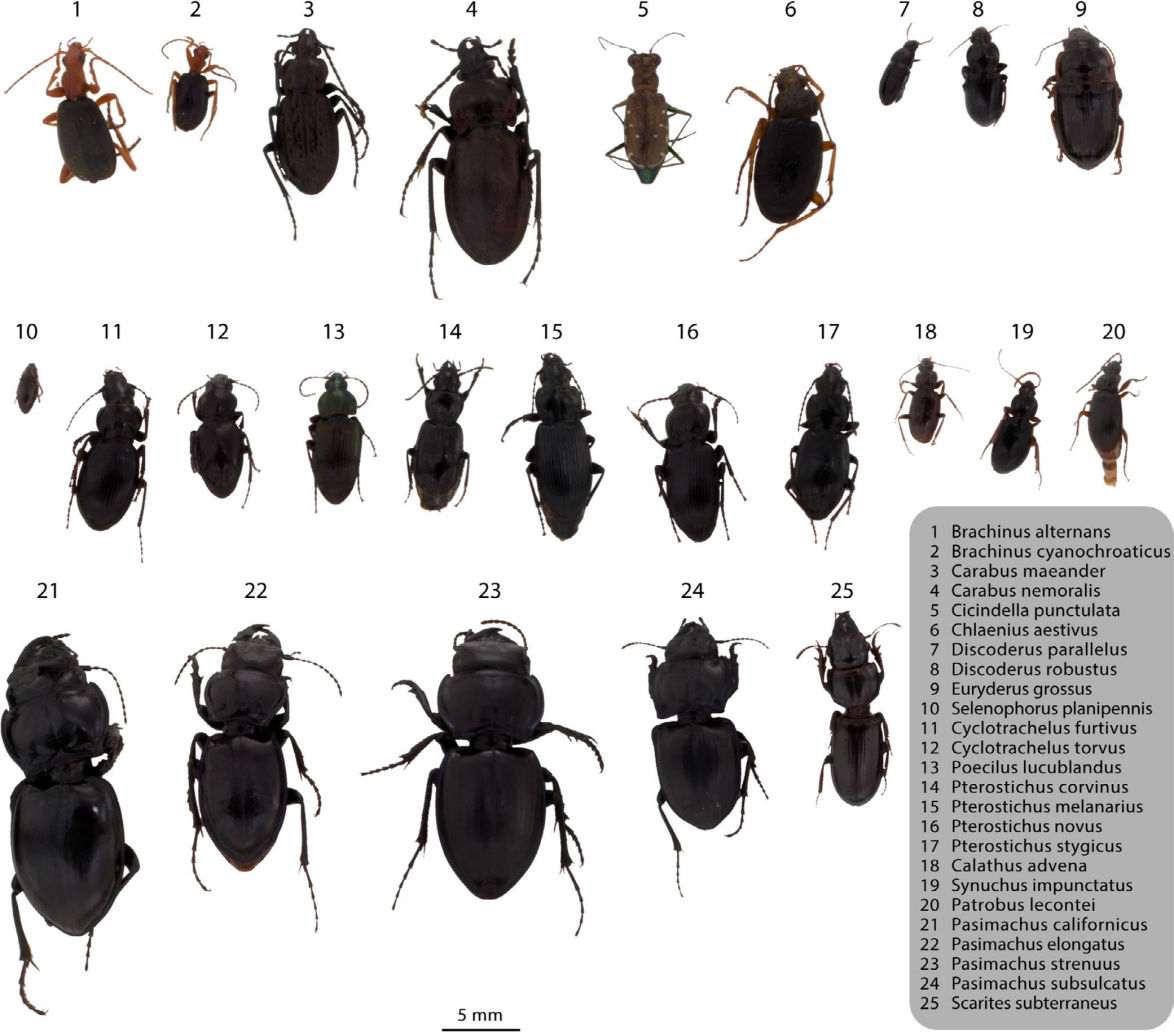
Collage of photographs of all Carabidae species used in the training dataset (*n* = 25). Specimens were cropped from their original photographs, and the background was removed. Relative scale of each specimen is conserved

Here, we develop a machine learning pipeline to identify batch samples of carabid specimens (family: Carabidae) collected by NEON pitfall traps. We contrast five machine learning algorithms of varying levels of complexity, ranging from k‐nearest neighbors (KNN) to artificial neural networks (ANN). Our objective is to present a proof‐of‐concept for developing a machine learning framework to identify bulk pitfall trap specimens from image data. We focused on the following questions: (a) Can machine learning models be used to identify carabid beetles from image data using feature vector methods? (b) Which models perform best? (c) Do hierarchical classifiers perform better than single‐level classifiers? and (d) Is machine learning a practical solution to current issues facing large‐scale insect monitoring efforts? We feel carabids are a good pilot case to answer these questions as they are abundant in the NEON dataset, diverse (~551 species identified by NEON to date), and relatively uniform in appearance (Figure 1), making them a challenging group to identify. Our pipeline is designed to be easily adaptable for other taxa, thus providing a possible machine learning solution for researchers that might not have access to the resources required for employing advanced deep learning models.

## 2 METHODS

### 2.1 Carabid collection

All specimens used in this project were collected by NEON in propylene glycol filled pitfall traps. For carabid beetle sampling protocols and procedures, refer to NEON Doc 014050vJ (Levan et al., 2015). All specimens were stored in 95% ethanol‐filled, 50 ml centrifuge tubes and shipped to our laboratory at the University of Oklahoma for imaging. As our goal was to develop these methods without pinning, we used ethanol stored specimens identified at NEON Domain Support Offices. We imaged 3,265 individual beetles from 580 tubes. All were collected in 2016. This represents the entire trapping season for 68 NEON trap arrays from 18 NEON sites (Table S1).

### 2.2 Imaging

To extract morphological data, we used a standardized digital imaging protocol. Beetles from an entire trapping event were batch‐imaged at the same time from above using an 8‐inch by 12‐inch section (20.3 cm by 30.5 cm) of a square foot nonreflective, white ceramic tile (Daltile© from Home Depot) on both their dorsal and ventral sides. We used a 50‐megapixel camera (Canon EOS 5Ds Model DS526521) and a 35 mm lens (Canon EF 35mm f/2 IS USM) to take raw images (i.e., in Canon.CR2 format) at a resolution of 27 pixels per mm. We used a white cloth light diffuser to reduce glare with lighting on four sides of the tile to reduce shadow. A total of 4–500 watt equivalent and 16–100 watt equivalent LED bulbs gave a light intensity at the center of the tile of 7,210 lx. The camera was mounted on a weighted boom stand (Diagnostic Instruments) and was not moved between images. All images were taken using the same camera settings (F22, 1/30 s, ISO 500) using a wired connection between the camera and computer running Canon EOS Utility 2 (Figure S1). To further standardize color, each image included an X‐rite ColorChecker Mini Classic (https://xritephoto.com/colorchecker‐classic) color reflection chart. Images had between 5 and 220 individuals (mean = 30.7 individuals), and creating both dorsal and ventral images took from 10 to 30 min per image, so we estimate less than one minute per individual.

Raw images were opened in Adobe Photoshop CC 2017 using the same color settings generated by the X‐Rite ColorChecker Passport Application (i.e., DNG file). We manually cropped the photograph to remove the tile edge and saved that file as an uncompressed.tiff file. We manually downscaled this.tiff file to a resolution of 12 pixels per mm. Using the FIJI implementation (Schindelin et al., 2012) of ImageJ (Schneider et al., 2012), we used two steps of manual color thresholding (Red then Black) to convert the images from color to binary, which produced a black/white binary image. Using the FIJI command “Analyze Particles,” we took morphological measurements of each individual region of interest (ROI, each an individual beetle). We used a macro to extract the RGB color values from the downscaled color images using the ROI map and the Color Histogram command in FIJI (see supplement for color extractor macro). This image postprocessing took approximately 20 min per image and did not vary with the number of individuals in the image.

### 2.3 Machine learning

The variables of the feature vector extracted using ImageJ fit into three broad categories: size, shape, and color. Size variables included specimen area, perimeter, width, height, and feret diameter (Ferreira & Rasband, 2012). Shape variables included circularity, aspect ratio, roundness, and solidity of the specimen (Ferreira & Rasband, 2012). Color variables were measured for each RGB color channel and included mean value, standard deviation, integrated density, skewness, kurtosis, and min/max value (Ferreira & Rasband, 2012). Each individual was photographed on their dorsal and ventral side, resulting in two values for each variable. In all, 68 descriptive variables were recorded and used for our training data. The variables collected through ImageJ greatly differ from what would normally be seen in an identification key used by humans. In humans, we tend to focus on categorical variables or simple continuous variables for identification, such as antennae segments, wing venation, or body length. In contrast, here we exclusively feed our models continuous variables, many of which would never really be used by humans (e.g., red color channel kurtosis). Code and raw data are available on the Open Science Framework (OSF) (Blair, 2020).

After the specimens had been processed and the image data extracted, we introduced the data to the machine learning pipeline (Figure S2). All steps in this pipeline were developed using R version 3.5.3 (R Core Team, 2019). First, species with 30 or fewer observations were separated from the dataset, as there would not be enough data for those species to robustly train the models. The dataset was then randomly split into separate training and testing subsets at a 70:30 ratio. This ratio maximized the amount of data that can be used for training while still leaving enough remaining samples to get robust measurements of model performance (Kho et al., 2017). Dataset splitting was repeated randomly 10 times, resulting in 10 training and testing subsets each (Figure S2).

After the data were split, it was standardized to meet the assumption of the models that the data are normally distributed (Hall, 2016). We standardized the data using the “center” and “scale” methods of the preProcess function in the R package caret (Kuhn et al., 2018), which normalized the predictor variables by subtracting the mean value and dividing by the standard deviation.

After standardizing, the data were ready to be used for training in a machine learning model. For this project, we decided to use five types of machine learning algorithms: K‐nearest neighbors (KNN; Cover & Hart, 1967), linear discriminant analysis (LDA; Mika et al., 1999), naïve Bayes (NB; Mika et al., 1999), random forests (RF; Ho, 1995), and artificial neural networks (ANN; Haykin, 2008).

*KNN*: To train the KNN model, we used the R package nabor (Elseberg et al., 2012). The model was trained at all integers of *k* between 1 and 25. The *k* value resulting in the highest top‐1 accuracy (Table 1) was used to measure all other performance metrics (Table 1, Figure S2).

**TABLE 1:** Definitions for the five measurements used to measure performance of our carabid classification models

Measurement	Definition
Top−1 accuracy	Measured as the accuracy of the model if only the prediction with the highest probability is counted.
Top−3 accuracy	Measured as the accuracy of the model if any of the predictions with the highest three probabilities matched the actual specimen name.
Precision	The macroaverage of the number of true positives divided by the number of true positives plus false positives for each taxonomic group.
Recall	The macroaverage of the number of true positives divided by the number of true positives plus false negatives for each taxonomic group.
F1 score	The macroaverage of the harmonic mean of precision and recall for each taxonomic group.

*LDA*: To train the LDA model, we used the R packages caret (Kuhn et al., 2018) and MASS (Venables & Ripley, 2002). No tuning parameters were modified for this model.

*NB*: To train the NB model, we used the R package e1071 (Meyer et al., 2017). No tuning parameters were modified for this model.

*RF*: To train the RF model, we used the R package randomForest (Liaw & Wiener, 2002). The model was trained at all integers of *mtry* (the number of available predictor variables at each node in the decision tree) between 1 and 10. The *mtry* value resulting in the highest top‐1 accuracy was used to measure all other performance metrics (Table 1, Figure S2).

*ANN:* To train the ANN model, we used the neuralnet R package (Fritsch et al., 2019). The model was trained at all integers of *hidden* (the number of neurons in each hidden layer) between 1 and 25. Only one hidden layer was used. The *hidden* value resulting in the highest top‐1 accuracy was used to measure all other performance metrics (Table 1, Figure S2).

To measure performance, each model predicted the taxa in the testing dataset. Performance was measured using five metrics listed in Table 1. In addition to these metrics, site‐specific performance was also measured using local species pools. We define local accuracy as the top‐1 accuracy when model classifications are filtered based on the known species pool for each sampling location (Figure S8). For example, if one site was only known to contain the carabid species *Chlaenius aestivus* and *Cyclotrachelus furtivus*, model classifications would be exclusively limited to those species. Species pools are determined based on occurrence in the NEON dataset. Local classifications were determined by reranking local species based on their original prediction probabilities output by the model. Local accuracy is only calculated at the species level.

For each model, we employed two classifiers: an hierarchical classifier and a single‐level classifier. In the hierarchical classifier, the models were trained and tested using the species‐level identification labels only. After the models were tested and species‐level classifications were made, we recorded performance measurements at all taxonomic levels based on the species predictions (Figure S4). For example, if the model classified a *Pasimachus strenuus* as *Pasimachus californicus*, it would be recorded as incorrect at the species level but correct at the genus level because both species belong to the genus *Pasimachus*. Likewise, if a *Scarites subterraneus* was misidentified as *Cyclotrachelus torvus*, it would be recorded as incorrect for every tested taxonomic level because they do not belong to the same subfamily (the highest taxonomic level used in this study). In the single‐level classifier, the models were trained and tested at all taxonomic levels, using the corresponding labels at each level (e.g., the genus‐level model was trained using generic names). This resulted in separate models that were used to make classifications within each respective taxonomic level only (e.g., the genus‐level model was only used to predict a specimen's genus; Figure S5).

Novel species classification was performed by testing the models on species that were not abundant enough to be included in the training dataset but belong to more common taxonomic clades at lower resolution (Figure S6). Due to these species not being included in the training dataset, they can be considered “novel” to the models, as the models will have no way of knowing they exist. For the hierarchical classifier, we forced the models to make a species‐level classification, but only recorded performance measurements at taxonomic levels where the novel species belonged to a common clade. For example, *Pterostichus trinarius* was too rare to be included in the training dataset but belongs to a common genus. If *P. trinarius* was classified as *Cyclotrachelus furtivus* by the hierarchical classifier, performance would not be measured at the species and group levels but would be measured at the genus level and above. In this example, the classification would be incorrect at the genus level, but correct at the subtribe level and above, as both species belong to the subtribe Pterostichini.

To compare our models to a successful general classification model, we also submitted 25 images (one per species in our training dataset) to iNaturalist to be identified using their automatic identification feature (Figure 1). Accuracy was measured for top‐1 and top‐3 predictions at the species and genus level.

### 2.4 Data description

The NEON carabid dataset contained a total of 3,270 individuals representing 64 species, 32 genera, 19 tribes, and 8 subfamilies (Figure S7). There were 398 trapping events (all in 2016) across 18 sites (latitudinal range: 29.7–47.2° N) with an average of 8.22 individuals per trap (Figure S8). The number of specimens collected from each of the 18 sites ranged from 10 to 567, with an average of 182 (*SD* = 161). After removing uncommon/unidentified species and splitting the entire dataset, training subsets ranged from 2029 to 2042 individuals and testing subsets ranged from 871 to 884 individuals. Variation in dataset size was due to the random sampling method used when splitting the datasets. The total number of classification categories in the training datasets ranged from 25 (species) to six (subfamily) after removing uncommon species from the NEON dataset. Classification categories were further reduced when model predictions were filtered using local species pools (Figure S3). The number of species in each local pool ranged from one to seven, with an average of three species. Sites with only one local species were not used when calculating local performance metrics, as they would be guaranteed to have perfect accuracy. This resulted in 14 sites used for calculating local performance metrics.

## 3 RESULTS

### 3.1 Performance – hierarchical classifier

**FIGURE 2:**
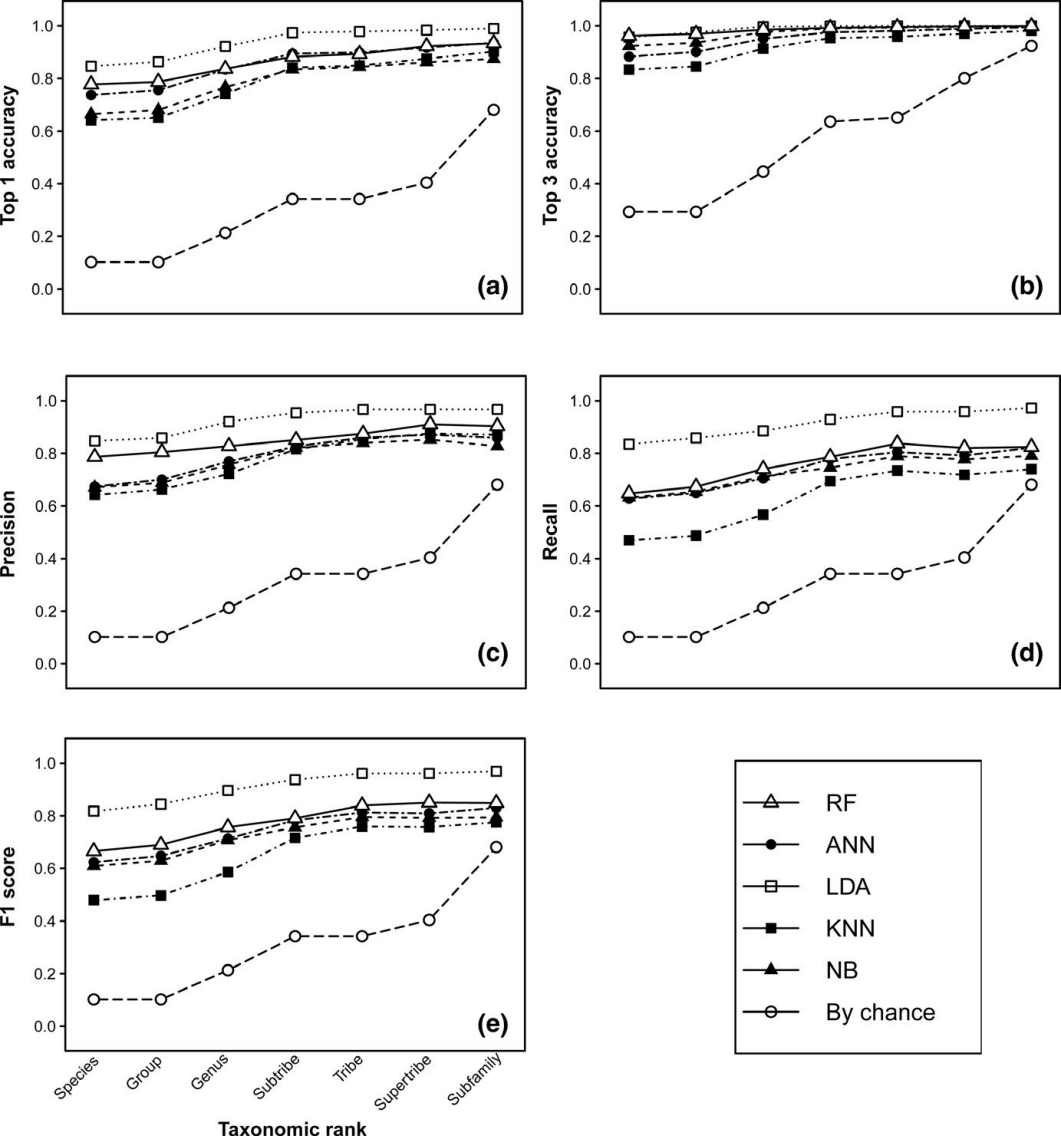
Carabidae machine learning performance plots for five algorithms across all tested taxonomic levels (Artificial neural network (ANN); Random Forest (RF); Linear discriminant analysis (LDA); K‐nearest neighbors (KNN); Naïve Bayes (NB); Expected performance if the model classified everything as the most common species (By chance). Results were taken as an average of 10 models for each algorithm. (a) Top‐1 Accuracy; (b) Top‐3 Accuracy; (c) Precision; (d) Recall; (e) F1 score

**FIGURE 3:**
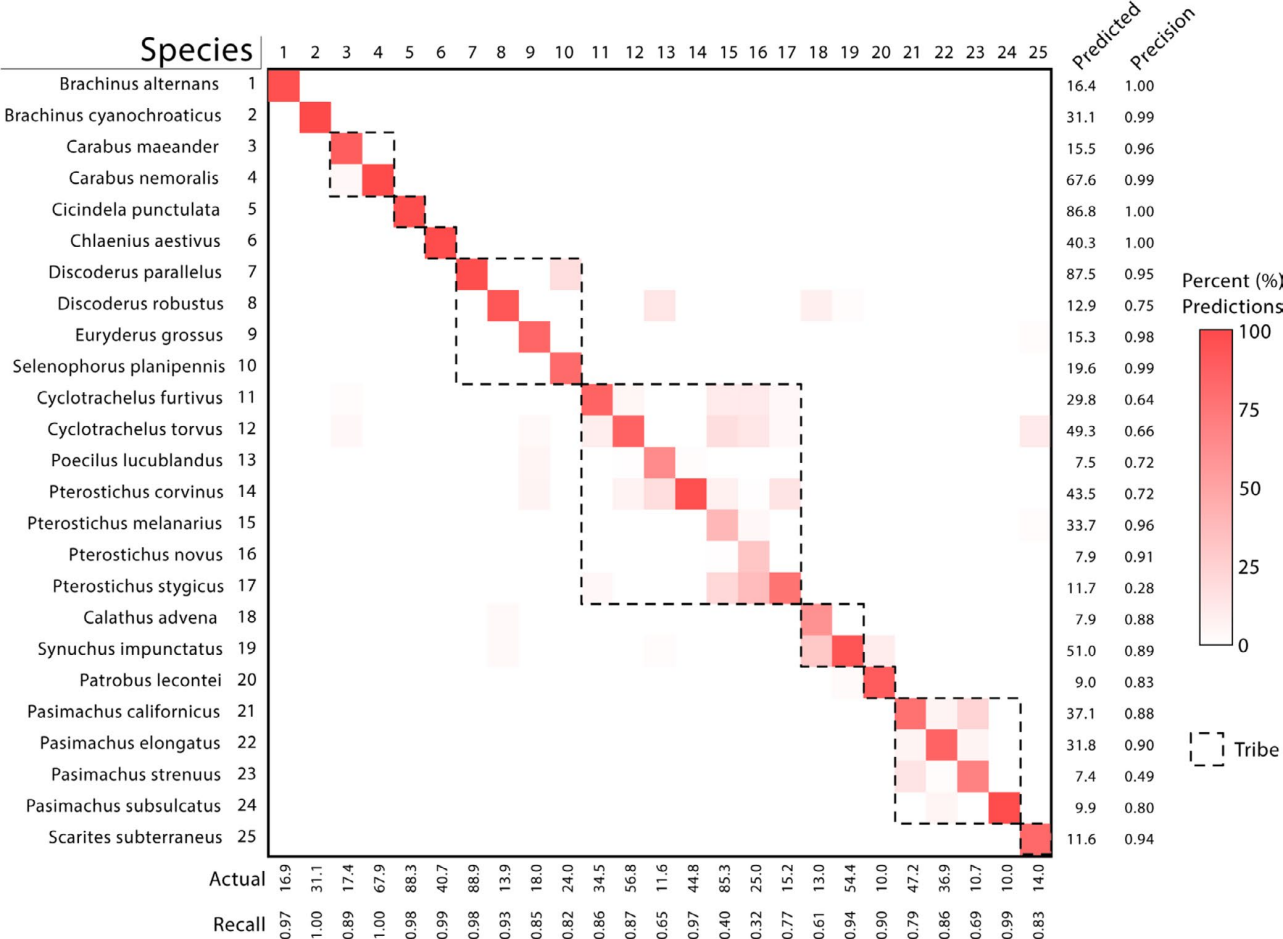
Linear discriminant analysis (LDA) Carabidae classification confusion matrix. Columns represent the actual observed species while rows represent what was predicted by the LDA model. The “Actual” row at the bottom of the graph indicates how many (on average) individuals of that species were present in the test datasets. The “Predicted” column indicates how many times (on average) each species was predicted by the LDA model. Results reflect an average of 10 LDA models. Box shading was applied as a percentage of the actual number of observations for each species. The hashed boxes indicate species from a single tribe

The LDA model had the best performance metrics in every category across all taxonomic levels and had an average top‐1 accuracy of 84.7% at the species level (Figure 2). Conversely, the KNN models generally performed most poorly, recording the lowest scores in 28 of 35 performance categories across all taxonomic levels (Figure 2). Since the model would not be able to identify rare species not included in the model at the species level, it can be assumed that our models would have a 0% accuracy when classifying those species. This would lower our original top‐1 accuracy of the LDA model to 75.4% when identifying all NEON carabids. Despite this, our models perform very well compared to iNaturalist's automatic identification feature. When tested on one image of each of our 25 Carabidae species, iNaturalist was 16% accurate (4/25) at the species level and 48% accurate (12/25) at the genus level (Table S2). Given that the LDA model delivered the highest performance metrics, we further investigated the model's performance sensitivity as a function of species prevalence and taxonomic relatedness. The precision recall and F1 score of the LDA models at the individual species level were positively correlated with species prevalence in the training dataset (Figure S9). Incorrect classifications at the species level were usually contained within the tribe (Figure 3).

For species‐level models, filtering model predictions using local species pools increased model accuracy. LDA had the highest local pool accuracy at 95.6%, while NB had the largest increase in accuracy when the local pool filter was applied (+25%; Figure S10). On average, model accuracy increased 16% when the local pool filter was applied. Each site had an average of 2.93 Carabidae species that were included in our models. The LDA model also reported 100% accuracy in species identification at the most sites (5 out of 14) (Figure S11).

Species that were removed from the training dataset may still belong to a common clade at a higher taxonomic level (e.g., genus). For these species, the models performed worse compared to predictions on species included in the training dataset (Figure S12). However, every algorithm still performed better than expected if predictions were made randomly, with naïve Bayes reaching an accuracy of 49.3% at genus.

### 3.2 Performance – single‐level classifier

The inverse relationship between taxonomic resolution and performance was also seen in the single‐level classifier whose relative performance was also consistent across algorithms (i.e., KNN performing worst, LDA performing best). However, the hierarchical classifier generally outperformed the single‐level classifier across all algorithms and taxonomic levels between group and subfamily (Figure S13).

Results were mixed when comparing the single‐level classifier to the hierarchical classifier at predicting the common clade of novel species. ANN, KNN, and RF generally had higher top‐1 accuracy when using the single‐level classifier, but only RF had an overall higher top‐3 accuracy when using this classifier (Figure S14).

## 4 DISCUSSION

Here, we detail the steps of a general pipeline for training machine learning algorithms to identify batch‐collected invertebrate specimens. The resulting best model (LDA), tested on a morphologically conservative beetle taxon, the carabids, correctly identified species 85% of the time when trained on a modest dataset of image‐based vectors. When identifying species included in the training dataset, our LDA model surpassed NEON’s commissioning requirements of 80% accuracy at the species level and approached the genus requirements of 95% accuracy. Moreover, if given site location information, the LDA model greatly surpasses both commissioning requirements, with a top‐1 species‐level accuracy of 95.6% (Figure S9). However, the “real‐world” accuracy of our models would be lower than this, as these measurements do not account for the error rate that would result from our model's inability to detect rare species not included in the training dataset. When taking this error rate into account, our LDA top‐1 accuracy decreases to 75%, while our local pool accuracy decreases to 85%. However, these numbers could be improved if we had more training data for these rare species, thus allowing them to be included in the models. In sum, our pipeline shows promise for developing accurate monitoring tools from small datasets of vouchered images, without considerable programming experience or expensive hardware.

Model performance improved with an hierarchical classifier, (Figure S13) suggesting that training models to the lowest possible taxonomic resolution and then working backwards is the optimal protocol. When predicting novel species to common clades, all models performed better than if classifications were made randomly (Figure S12). Although these models would never be able to classify a novel species to the species level, our results show they still can be useful at predicting novel species to higher taxonomic levels included in the training dataset. Our results comparing hierarchical versus single‐level novel species classification are mixed, as there was no consensus among algorithms in favor of either method (Figure S14). Given the superior performance and convenience of the hierarchical classifier, we see no reason to use a single‐level classifier in favor of an hierarchical classifier.

Incorporating known species ranges improves model predictions, but prevents the ability to detect species that have expanded their ranges. One solution may be to include contextual metadata as part of the image feature vectors (Terry et al., 2020). Contextual metadata could include primary metadata such as location and date, as well as secondary metadata (if collected from external data sources) such as weather and habitat type (Terry et al., 2020). The models can then weigh contextual metadata against image features when making a prediction. This leaves open the possibility of predicting locally novel species while still using location information to improve accuracy.

Another way that our pipeline may be useful is in the simultaneous analysis of multiple specimens. Most insect monitoring efforts, like NEON, collect insects in bulk. By using high‐resolution images of a single trapping event, instead of individually imaging each specimen as in other such studies (Joutsijoki et al., 2014; Kho et al., 2017; Larios et al., 2008; Mayo & Watson, 2007), we achieve similar top‐1 accuracies range from 75% to 95%, with total number of classes ranging from 3 to 35. Another study similar to ours (Ärje et al., 2020) uses robotics and more complex machine learning algorithms with the goal of increasing invertebrate sorting efficiency. While this study produced a higher average accuracy than ours (98%), they also tested fewer species (9) across a broader taxonomic range (terrestrial arthropods). Additionally, methods such as those used by Ärje et al. may not be suitable for large‐scale insect monitoring, as they require specialized equipment, have limited throughput (1 specimen at a time), and would require far more image data storage (~50 images per specimen compared to our two images (dorsal and ventral) for dozens of specimens), which would become increasingly problematic the larger a monitoring program gets. Additionally, our use of pre‐extracted feature vectors allows users to better understand which variables are important to our model's performance compared to more opaque methods like convolutional neural networks. For algorithms like random forests, such variable importance can be determined using a single function (Liaw & Wiener, 2002). Moreover, we show that bulk collections like NEON’s, which may be lower quality as they amass samples over two weeks in propylene glycol, still provide high‐resolution IDs using image extracted feature vectors. Compared to more traditional, human‐exclusive identification pipelines, the imaging process in our pipeline may still present a potential bottleneck. However, such a bottleneck may be alleviated using additional automation such as a conveyor system (Sweeney et al., 2018), and the imaging in itself can provide other benefits, such as creating a digital biorepository (Nelson & Paul, 2019). Overall, our methods show great promise for efficient continental scale insect monitoring.

Finally, we show that building smaller, more specialized models, especially for noncharismatic insects like carabid beetles, is a better option than relying on large‐scale citizen science‐based tools like iNaturalist. The poor performance of iNaturalist's identification tool (Table S1) is unsurprising, as iNaturalist is designed to identify thousands of species with an image database mostly crowdsourced from citizen scientists. As such, its models will be biased toward charismatic and abundant species that people are likely to photograph. This causes less “exciting” but nonetheless ecologically important species (like many of our carabids) to go ignored by their models. Tools such as iNaturalist serve an important purpose and do well at achieving their intended functionality. However, these tools should not be considered a catch‐all solution for species identification problems. We included the iNaturalist comparison to illustrate that, when solving a problem using machine learning, it is important to ensure the selected solution is designed to solve the particular problem.

For researchers that wish to adopt our methodology, we see two primary use cases. The first use case we envision are researchers want to use our models to classify carabid beetles included in this study. In this case, researchers should follow our image data collection and classification protocol, and additional model training may not be needed, as they can use our pretrained models (Blair, 2020). The other use case we envision is researchers that want to adopt our general methodology for a different taxonomic group. In this case, our pretrained models would serve no function, but our general methodology would still be useful. The versatility of machine learning algorithms should allow anyone that follows our image data extraction protocols to train their own classification models using our training protocols, regardless of what the image subjects are. Users of our pipeline may also wish to use machine learning algorithms other than the five chosen for this study. Such a change would only require some simple edits to our code, particularly for machine learning algorithms included in caret that support multiclass classification (Kuhn et al., 2018). Finally, pre‐extracted feature vectors result in datasets that contain useful measurements such as size and color data that may be used for other analyses beyond machine learning classification.

A primary goal of large‐scale insect monitoring projects such as NEON is to collect reliable occurrence and abundance data. However, due to the scale of these projects, it is difficult to collect such data in a timely manner. Given the current state of global insect populations, it is essential that we collect as much data as possible to aid efforts to conserve insect biodiversity (Thomas et al., 2019). We propose that the use of machine learning algorithms will increase efficiency of identification pipelines, leading to a greater flow of data that can help us understand these macroecological trends.

## CONFLICT OF INTEREST

Jarrett Blair is the CEO and cofounder of Luna ID Inc., a mobile application development company that specializes in developing apps to identify insects from images using machine learning. However, the contents of this manuscript are purely academic, and the methods herein have no commercial purpose.

## AUTHOR CONTRIBUTIONS

**Jarrett Blair:** Conceptualization (equal); Data curation (lead); Formal analysis (lead); Investigation (lead); Methodology (equal); Visualization (equal); Writing‐original draft (lead); Writing‐review & editing (equal). **Michael D. Weiser:** Conceptualization (equal); Data curation (equal); Funding acquisition (equal); Methodology (equal); Writing‐review & editing (equal). **Michael Kaspari:** Conceptualization (equal); Funding acquisition (equal); Project administration (lead); Writing‐review & editing (equal). **Cameron Siler:** Conceptualization (equal); Funding acquisition (equal); Visualization (equal); Writing‐review & editing (equal). **Matthew Miller:** Conceptualization (equal); Funding acquisition (equal); Writing‐review & editing (equal). **Katie E. Marshall:** Conceptualization (equal); Formal analysis (equal); Funding acquisition (equal); Investigation (equal); Methodology (equal); Resources (equal); Supervision (lead); Visualization (equal); Writing‐review & editing (equal).

## Supporting information

Supplementary Material

## Data Availability

All image data, ImageJ scripts, and R code are available on OSF (https://doi.org/10.17605/OSF.IO/2AEY7).
