# Supplementary material for: Robust and simplified machine learning identification of pitfall trap‐collected ground beetles at the continental scale
Source: Ecol Evol. 2020 Nov 11;10(23):13143–53. doi: 10.1002/ece3.6905 (PMC7713910; doi:10.1002/ece3.6905)
Supplement: Supplementary file 1 — Supplementary Material [file ECE3-10-13143-s001.docx]

**Supplemental**

**Figures**

**
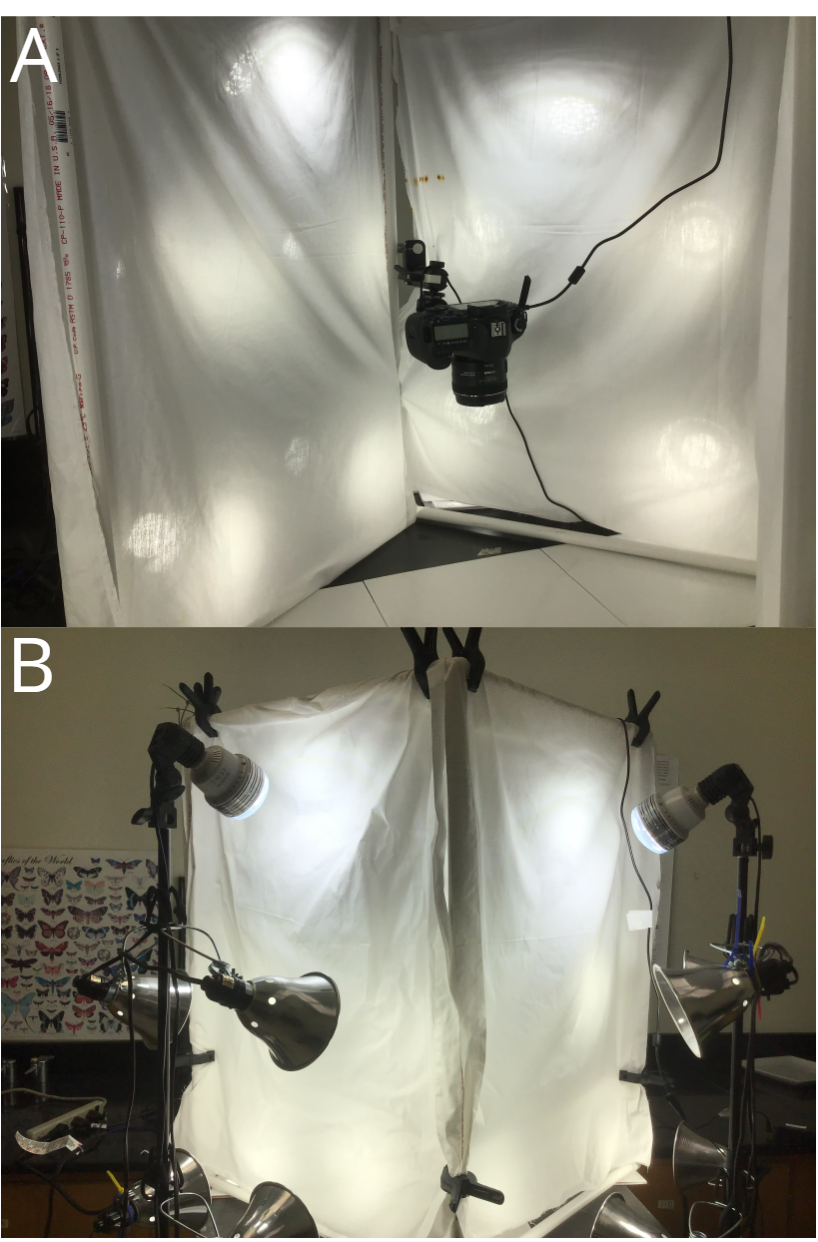
**

Figure S1. Imaging setup. (A) Canon EOS 5Ds Model DS526521 positioned above imaging plate. (B) One half of the lighting set up. A total of 16 100-watt equivalent and four 500-watt equivalent LED bulbs were placed outside of a white cloth light diffuser. Specimens were photographed inside the light diffuser.


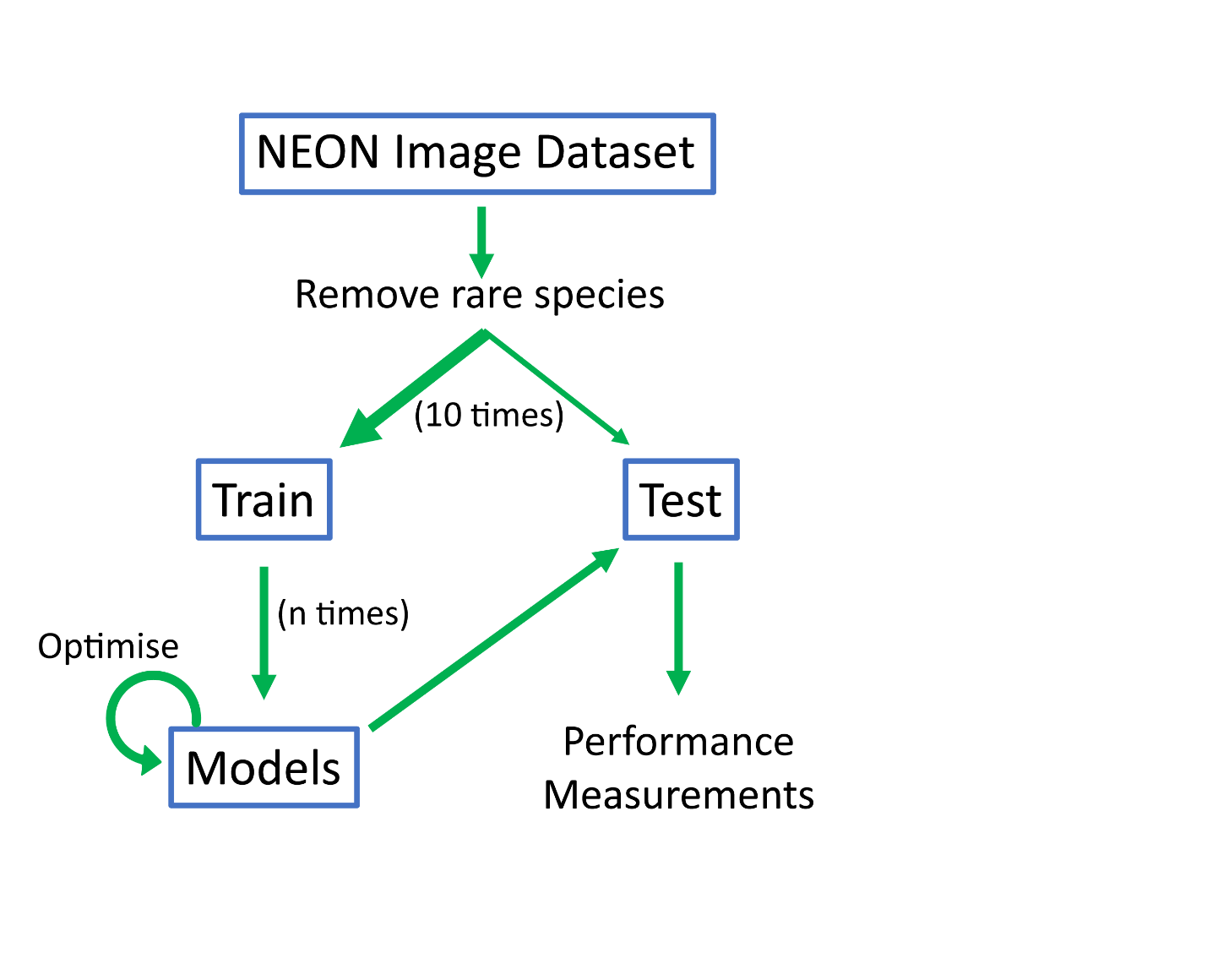


Figure S2: Flow diagram showing how our machine learning pipeline turns image data inputs into model performance outputs. First, rare species (<30 observations) are removed before the dataset is split into training and testing sets 10 times at a ratio of 70:30 (indicated by arrow weight). For iterative algorithms like k-nearest neighbours, random forests and artificial neural network, the models are trained and optimized on the training data n times. The value of n varies depending on the model and tuning parameter being used. After optimisation, the models make predictions on the test data, and performance is measured based on these predictions.


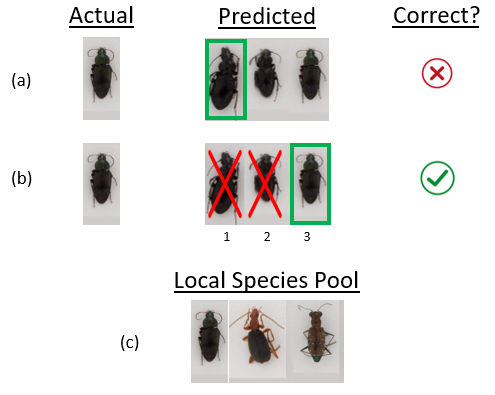


Figure S3: Mock example illustrating how a local filter can increase classification accuracy. (a) *Poecilus lucublandus* is incorrectly classified as *Cyclotrachelus furtivus*, with *Cyclotrachelus torvus* and *P. lucublandus* being the second and third classifications, respectively. (b) The *C. furtivus* and *C. torvus* classification are removed by comparing the classifications with the local species pool (c). At this example location, the local species pool consists of *P. lucublandus*, *Brachinus alternans* and *Cicindella punctulata*. This leaves *P. lucublandus* as the new top classification, which is correct.


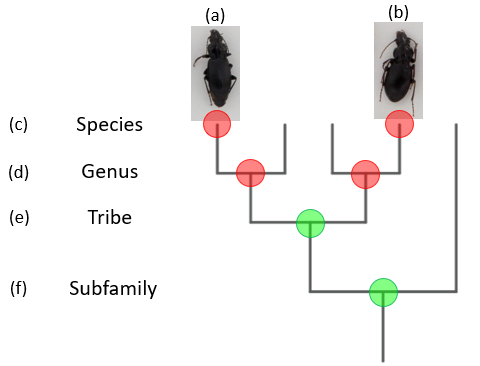


Figure S4: Mock example illustrating how accuracy of the ontological classifier is determined at different taxonomic levels. In this example, a Cyclotrachelus furtivus (a) specimen is classified by the model as Pterostichus melanarius (b). The classification is deemed incorrect at the species (c) and genus (d) level, but is correct at the tribe (e) level because both species belong to the Pterostichini tribe. The classification then guaranteed to be correct at all subsequent levels (f).


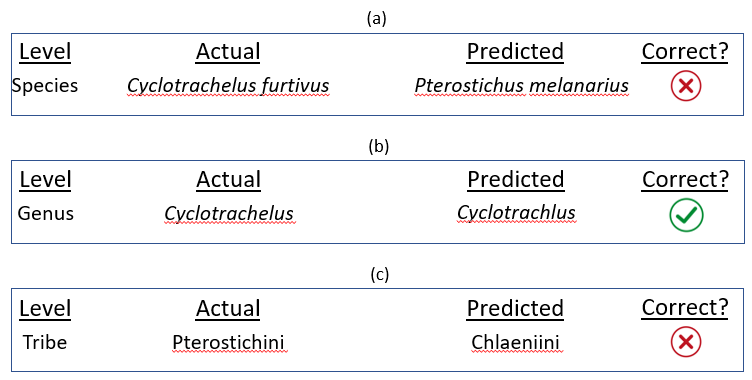


Figure S5: Mock example illustrating how accuracy of the single-level classifier is determined at different taxonomic levels. At the species level (a), Pterostichus melanarius is incorrectly classified as Cyclotrachelus furtivus. At the genus level (b) the classification can be correct because a separate model is trained for each taxonomic level, which means the genus level classification is not dependent on the species level classification. However, this may also have negative consequences as classifications at subsequent taxonomic levels (e.g. tribe (c)) are not guaranteed to be correct even if previous levels were correct.


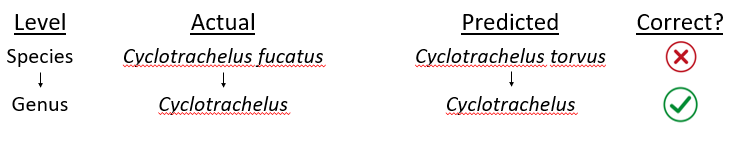


Figure S6: Mock example illustrating how the ontological classifier classifies novel species. A novel species is defined as a species that had <30 observations in the NEON dataset and thus were excluded from the training dataset. In this example, Cyclotrachelus fucatus is classified as Cyclotrachelus torvus at the species level. Cyclotrachelus fucatus was not included in the training dataset, so the species level classification will always be incorrect as the model has no way of knowing C. fucatus exists. However, the classification can still be correct at subsequent levels, as C. fucatus shares a genus with other species in the training dataset.


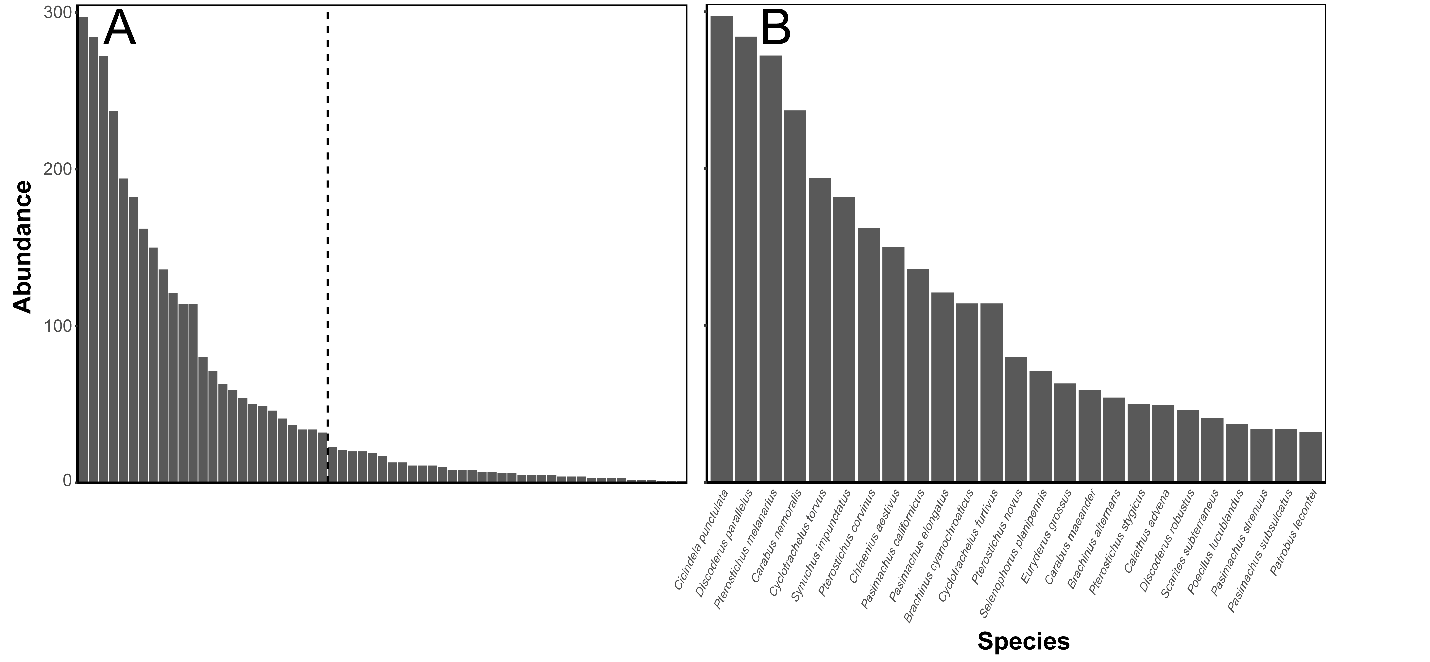


Figure S7: (A) Rank abundance of Carabidae species in the NEON dataset. Each bar represents one species. Data was collected for 63 species, 25 of which were included in the models. The dashed line indicates the cut off point for species to be included in the models (> 30 observations). Unidentified individuals are not included. (B) Rank abundance of species included in the training datasets, with species names along the x-axis.
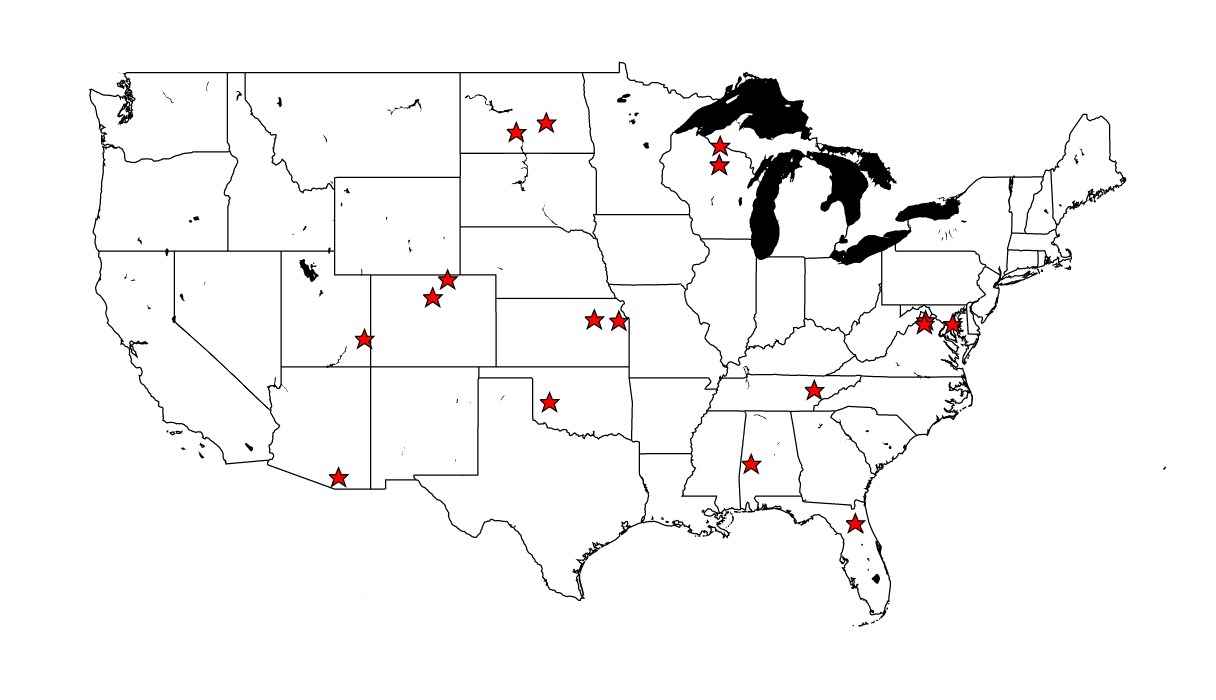


Figure S8: The 18 NEON sampling locations where the carabid specimens used in this study were collected. Each location is represented on the map by a red star.


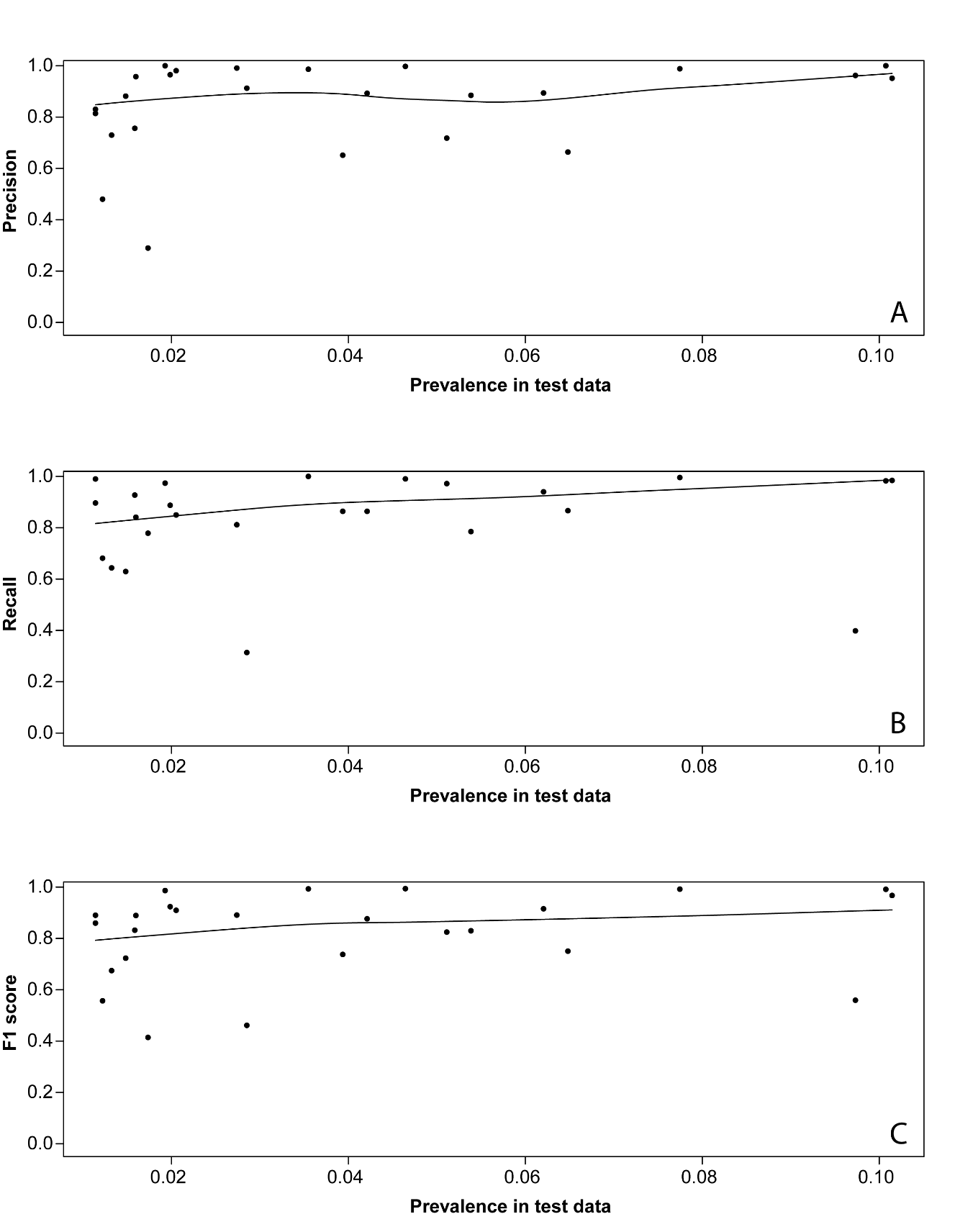


Figure S9: (A) Precision, (B) recall & (C) F1 score measurements for the linear discriminant analysis (LDA) models trained to classify Carabidae species from image data. Recorded measurements are an average across 10 LDA models. Each point represents one species (n = 25). Performance measurements for each species are plotted against the prevalence of that species in the test data. A local regression curve is represented as a line on each plot.


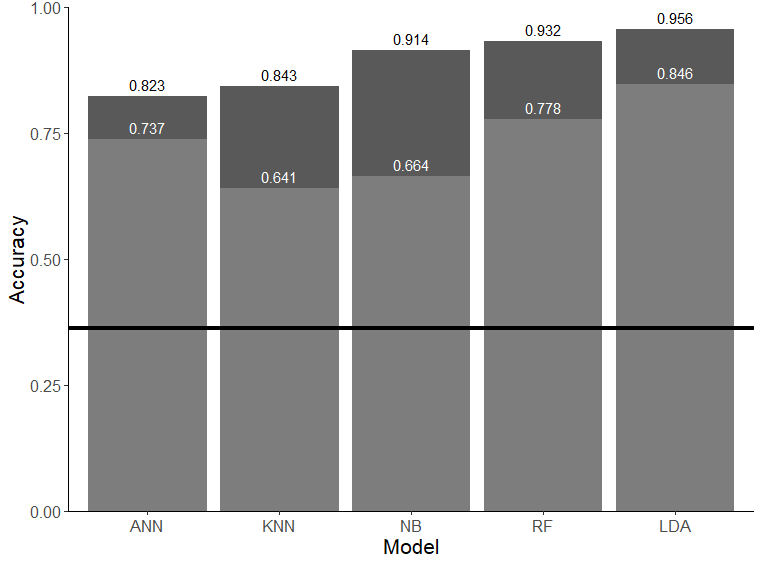


Figure S10: Improvement of top-1 Carabidae image data classification accuracy for five machine learning algorithms after filtering for local pool. Predictions were restricted to species that were known to exist at each NEON collection site (“local pool accuracy”). Only sites with >1 local species in the training datasets were included in this analysis (n =14). Light grey bars represent top 1 accuracy without the local species pool restriction. The horizontal line indicates the expected accuracy if predictions for each site were made randomly. Artificial neural network (ANN); Random forest (RF); Linear discriminant analysis (LDA); K-nearest neighbours (KNN); Naïve Bayes (NB).


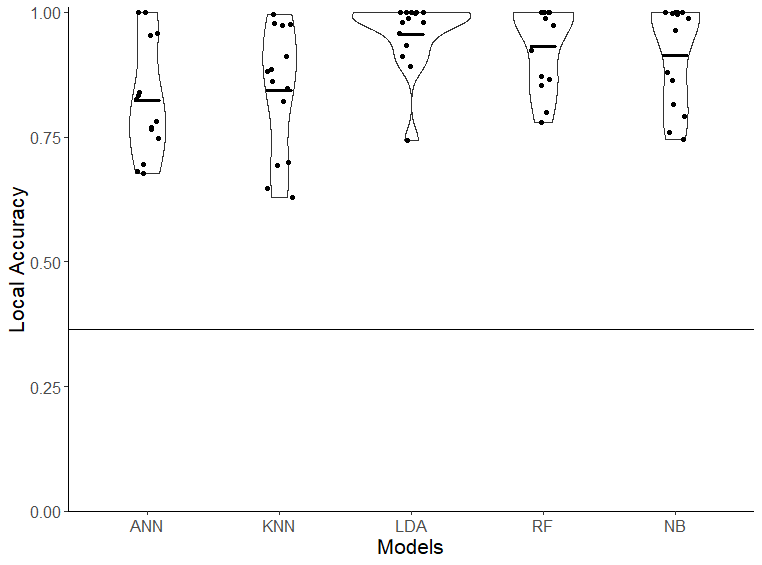


Figure S11: Accuracy of Carabidae species identification for each NEON collection site across the five trained machine learning models (Artificial neural network (ANN); Random forest; Linear discriminant analysis (LDA); K-nearest neighbours (KNN); Naïve Bayes (NB)). Each point represents one site (n = 14). The small horizontal lines represent the mean accuracy across all sites for each model. The large horizontal line represents the average expected accuracy if predictions were made randomly at each site (36.4%). The average number of tested species per site was 3.14.
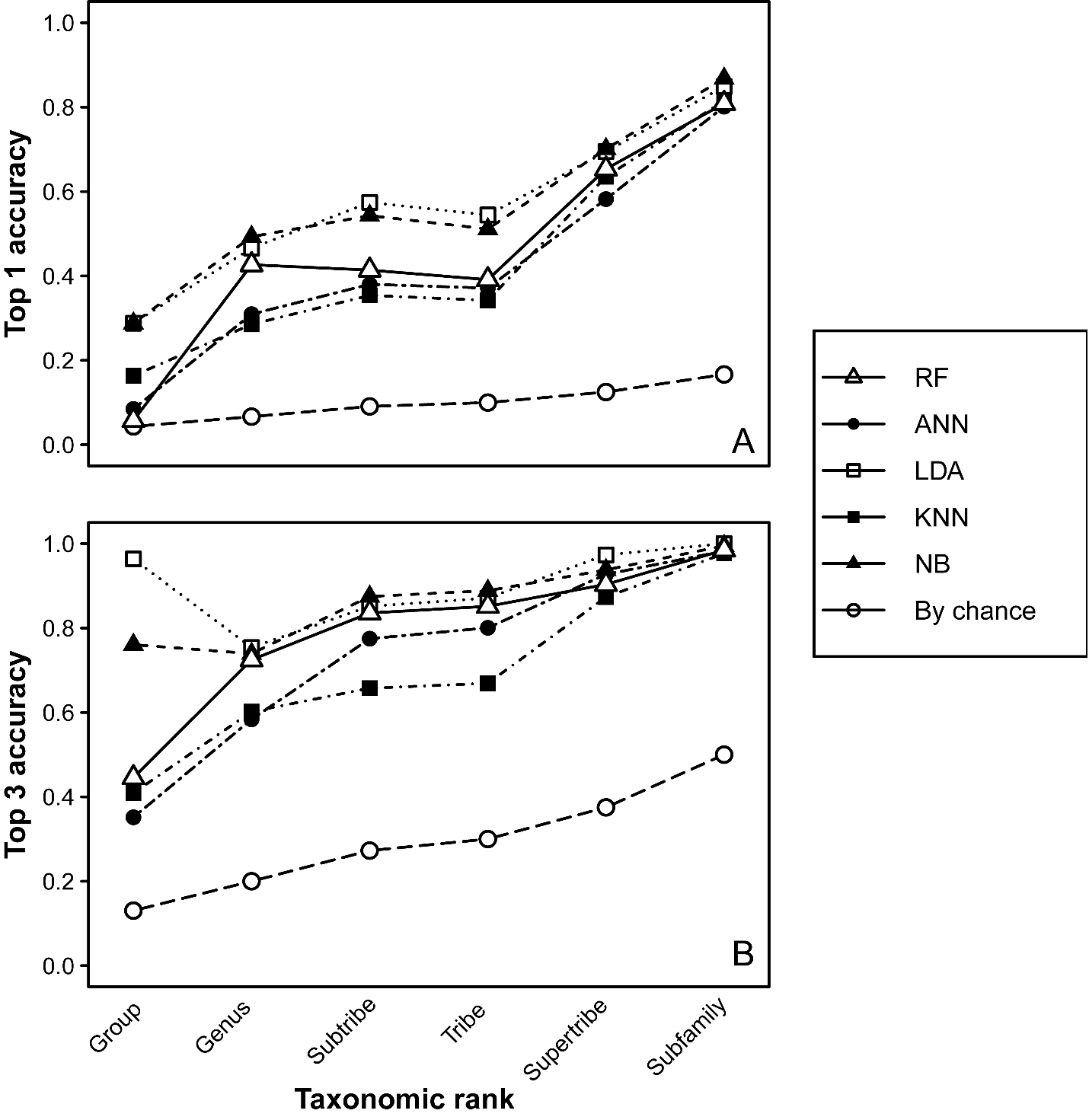


Figure S12: Carabidae machine learning performance plots for five algorithms across all tested taxonomic levels when tested on novel species belonging to clades included in the training dataset (Artificial neural network (ANN); Random Forest (RF); Linear discriminant analysis (LDA); K-nearest neighbours (KNN); Naïve Bayes (NB); Expected performance if predictions were made randomly (By-chance)). Results were taken as an average of 10 models for each algorithm. (A) Top-1 Accuracy; (B) Top-3 Accuracy.


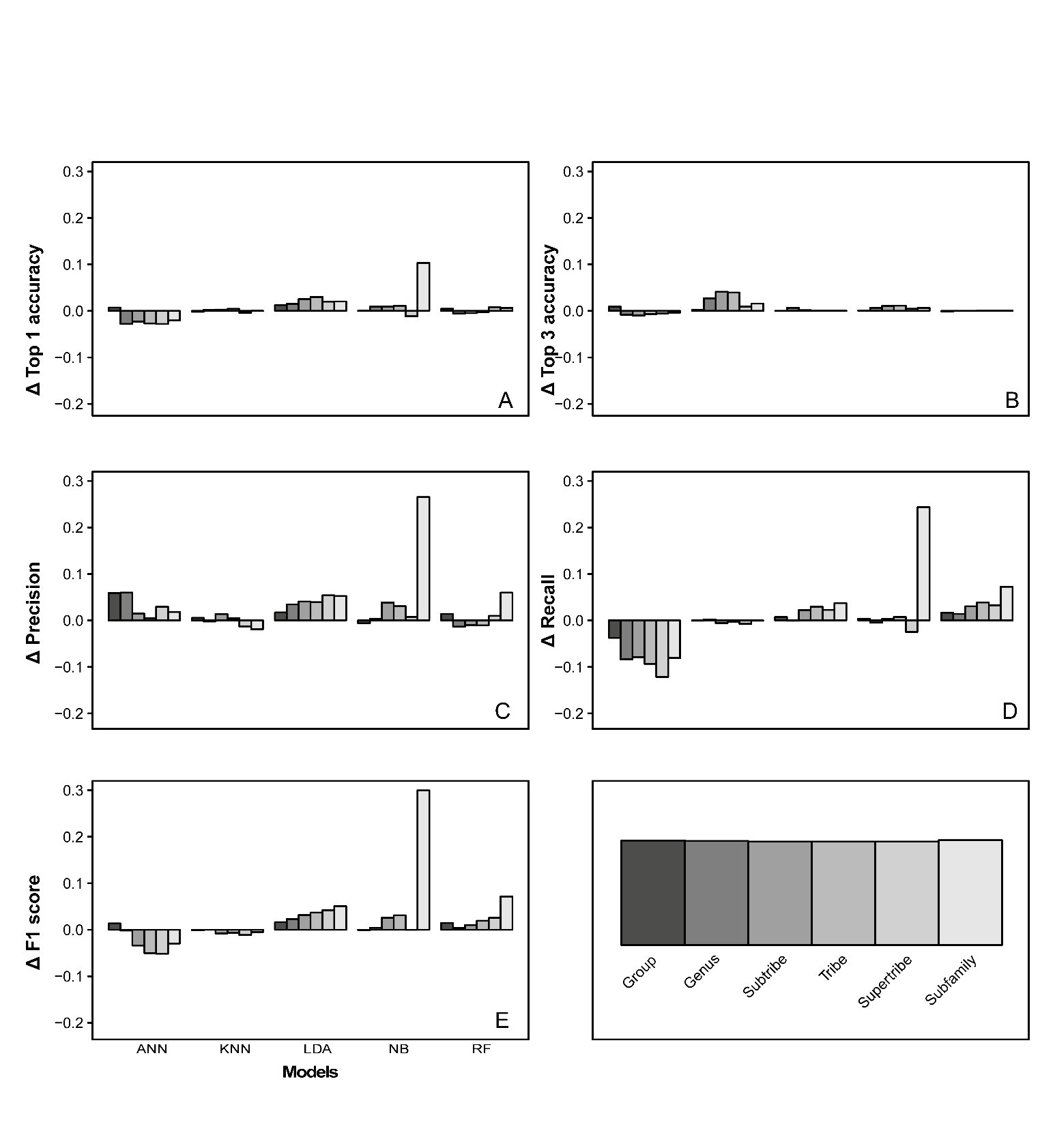


Figure S13: Carabidae classification performance differences between ontological and single-level machine learning classifiers. Five machine learning algorithms were used: Artificial neural network (ANN); Random Forest (RF); Linear discriminant analysis (LDA); K-nearest neighbours (KNN); Naïve Bayes (NB). Differences in performance were measured from group to subfamily. Difference values indicate the improvement by ontological model performed compared to the single-level model for each given algorithm, taxonomic level and performance metric. (A) Top-1 Accuracy; (B) Top-3 Accuracy; (C) Precision; (D) Recall; (E) F1 score.


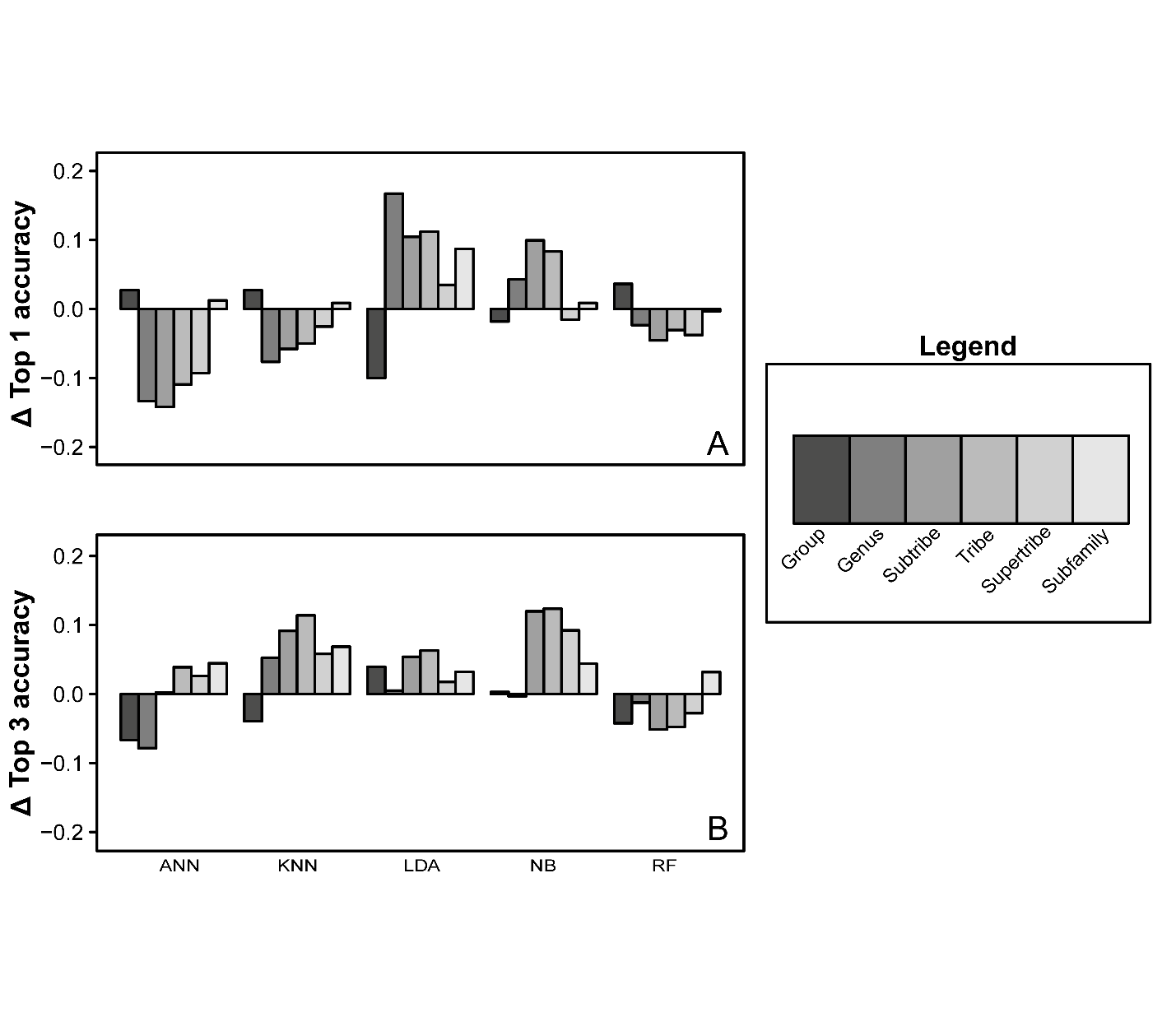


Figure S14: Carabidae classification performance differences between ontological and single-level machine learning classifiers when tested on novel species belonging to clades included in the training dataset. Five machine learning algorithms were used (Artificial neural network (ANN); Random Forest (RF); Linear discriminant analysis (LDA); K-nearest neighbours (KNN); Naïve Bayes (NB)). Differences in performance were measured between group and subfamily. Difference values indicate the improvement by ontological model performed compared to the single-level model for each given algorithm, taxonomic level and performance metric. (A) Top-1 Accuracy; (B) Top-3 Accuracy.

**Tables**

Table S1: The 18 NEON sites used in this study representing 9 habitat types (Dominant NCLD Classes).

| NEON Site | Dominant NCLD Class(es) |
| --- | --- |
| Blandy Experimental Farm | Deciduous Forest,  Pasture/Hay |
| Central Plains Experimental Range | Grassland/Herbaceous |
| Dead Lake | Evergreen Forest,  Woody Wetlands |
| Konza Prairie Biological Station | Deciduous Forest,  Grassland/Herbaceous |
| Moab | Evergreen Forest,  Shrub/Scrub |
| Niwot Ridge Mountain Research Station | Evergreen Forest,  Grassland/Herbaceous |
| Northern Great Plains Research Laboratory | Grassland/Herbaceous |
| Klemme Range Research Station | Grassland/Herbaceous,  Shrub/Scrub |
| Oak Ridge | Deciduous Forest,  Evergreen Forest,  Pasture/Hay |
| Ordway-Swisher Biological Station | Emergent Herbaceous Wetlands,  Evergreen Forest,  Woody Wetlands |
| Smithsonian Conservation Biology Institute | Deciduous Forest,  Evergreen Forest,  Pasture/Hay |
| Smithsonian Environmental Research Center | Cultivated Crops,  Deciduous Forest |
| Santa Rita Experimental Range | Shrub/Scrub |
| Steigerwaldt Land Services | Deciduous Forest,  Mixed Forest,  Woody Wetlands |
| Treehaven | Deciduous Forest,  Evergreen Forest,  Mixed Forest,  Woody Wetlands |
| The University of Kansas Field Station | Deciduous Forest,  Pasture/Hay |
| UNDERC | Deciduous Forest,  Mixed Forest,  Woody Wetlands |
| Woodworth | Emergent Herbaceous Wetlands, Grassland/Herbaceous |

Table S2: Accuracy measurements for Carabidae species photo identification using iNaturalist’s automatic identification feature. iNaturalist was tested using images of 25 carabid species, with one photo per species. Predictions of an incorrect species but correct genus counted positively towards genus accuracy. Minimum, maximum and mean number of observations were calculated using the total number of recorded observations of each species/genus on iNaturalist.

|  | Top 1 | Top 3 | Min. Obs. | Mean Obs. | Max Obs. |
| --- | --- | --- | --- | --- | --- |
| Species | 0.16 | 0.2 | 0 | 191.2 | 1629 |
| Genus | 0.48 | 0.56 | 6 | 3412.2 | 18429 |
